# The Ubiquitin Ligase Praja1 Reduces NRAGE Expression and Inhibits Neuronal Differentiation of PC12 Cells

**DOI:** 10.1371/journal.pone.0063067

**Published:** 2013-05-22

**Authors:** Jan Teuber, Bettina Mueller, Ryoji Fukabori, Daniel Lang, Anne Albrecht, Oliver Stork

**Affiliations:** 1 Department of Genetics and Molecular Neurobiology, Institute of Biology, Otto-von-Guericke-University Magdeburg, Magdeburg, Germany; 2 Center for Behavioural Brain Sciences, Magdeburg, Germany; CNRS UMR7275, France

## Abstract

Evidence suggests that regulated ubiquitination of proteins plays a critical role in the development and plasticity of the central nervous system. We have previously identified the ubiquitin ligase Praja1 as a gene product induced during fear memory consolidation. However, the neuronal function of this enzyme still needs to be clarified. Here, we investigate its involvement in the nerve growth factor (NGF)-induced differentiation of rat pheochromocytoma (PC12) cells. Praja1 co-localizes with cytoskeleton components and the neurotrophin receptor interacting MAGE homologue (NRAGE). We observed an enhanced expression of Praja1 after 3 days of NGF treatment and a suppression of neurite formation upon Praja1 overexpression in stably transfected PC12 cell lines, which was associated with a proteasome-dependent reduction of NRAGE levels. Our data suggest that Praja1, through ubiquitination and degradation of NRAGE, inhibits neuronal differentiation. The two murine isoforms, Praja1.1 and Praja1.2, appear to be functionally homologous in this respect.

## Introduction

Differentiation of neuronal and non-neuronal cells occurs in interplay of intrinsic cellular programmes with signals from diffusible factors, matrix components and cell-to-cell interactions. Evidence has accumulated that ubiquitination and related processes play an active and critical role with regard to this interaction [1]. Expanding the classical view of ubiquitination as a regulator of protein half-life, signalling pathways have been identified that employ either monoubiquitination to control processes like intracellular trafficking and transcriptional regulation or polyubiquitination to target signalling molecules for proteasomal degradation during cellular differentiation. These processes may be particularly important in the developing and adult nervous system, which is characterized by a high degree of cellular differentiation and structural complexity. In fact, an involvement of polyubiquitination has been observed during the generation and modification of synaptic connections [2], [3], while genetic disruption of the ubiquitin ligases parkin and UBE3A have been implicated in severe neurological disorders, including Parkinson's disease [4], [5], Angelman syndrome [6], [7], or Fragile X Associated Tremor/Ataxia Syndrome [8]. The E3 ubiquitin ligase Praja1 (Sanskrit for “birth” or “development”) is a candidate for the control of neuronal development and plasticity in the nervous system. Praja1, which is expressed in the cytosol of hepatocytes in liver explants, has initially been identified as a gene related to liver development [9]. However, sequence similarity to Neurodap1 [10] and prominent expression in the brain also indicate an involvement in nervous system function [9], [11]. Furthermore, deletion of the region harbouring the PJA1 gene has been observed in patients with craniofrontonasal syndrome and may be associated with mild learning disabilities [12]. Several targets of Praja1-mediated polyubiquitination have already been identified, including the class II melanoma antigen (MAGE) family member NRAGE (neurotrophin receptor associated MAGE homologue), Smad3 and polycomb repressive complex 2 [13]–[15]. NRAGE (named Dlxin-1 in mouse and MAGE-D1 in human) may be of particular relevance for neuronal development; it is a multifunctional signalling molecule involved in – among others – neurotrophin (via p75^NTR^) and bone morphogenetic protein (BMP) signalling, as well as in UNC5H1 mediated cell adhesion, all of which are involved and appear to interact in neuronal differentiation [16]–[22]. NRAGE is highly expressed in the developing and adult nervous system, often but not exclusively together with p75^NTR^ [23], [24]. NRAGE has been shown to be pro-apoptotic in various cell types [24]–[27] and to be involved in the neuronal differentiation of pheochromocytoma (PC12) cells [28], [29]. PC12 cells endogenously express the NRAGE activator p75^NTR^ [24], which is known to mediate NGF-signalling in cell survival, differentiation and cell death [18], [24]. Praja1 binds to the necdin homology domain of NRAGE and – less efficiently – to necdin itself, leading to ubiquitination and proteasomal degradation of NRAGE and to a modulation of Msx2 and Dlx5-dependent transcription [30]. Control of NRAGE expression and activity through Praja1 may thus provide an important mechanism for controlling neuronal differentiation.

We tested this hypothesis and investigated the role of Praja1 in NGF-induced differentiation of PC12 cells. Two validated transcript variants of mouse *praja1* (praja1.1, NM_001083110.1 and praja1.2, NM_008853.3) were used, that code for two isoforms, referred to as Praja1.1 and Praja1.2, with a predicted molecular weight of 64 kD and 44 kD, respectively. Our data demonstrate the induction of Praja1 during neuronal differentiation, its intracellular localization and co-localization with NRAGE, and the Praja1-mediated reduction of NRAGE expression levels and of neurite outgrowth.

## Materials and Methods

### mRNA expression analysis

Isoform-specific gene expression was analysed with quantitative “real-time” polymerase chain reaction using FAM-labelled probes (custom TaqMan expression assays, Applied Biosystems, Foster City CA/USA) and a mouse-II-cDNA-panel (BD Bioscience, Paolo Alto CA/USA). Primers for praja1.1 (5′-GAGGAACCGGTGGTGAGA-3′ and 5′-AAAAACACTTTGGGTTTCATGCAGT-3′) were tagged with the FAM-reporter 5′-TTGGAGTCGCCACATTC-3′, and for praja1.2 (5′-CTGGCTGTTGAGAGTGAGGAT-3′ and 5′-CCTCAGCATCGGCAGCAT-3′) with the FAM-reporter 5′-CCCGCCACCTGGAATA-3′. For detection of the housekeeping gene phospho-glycerate kinase (PGK), we used assay Mm00435617_m1 (TaqMan gene expression assay, Applied Biosystems). For amplification and real-time quantification, samples were uracil-N-glycosylated for 2 min at 50°C before being denatured for 10 min at 95°C and amplified with up to 50 cycles of 15 s at 95°C and 1 min at 60°C. Typical quantification was performed within a range of 25 to 35 cycles. However, detection of rare splice forms occasionally required 40 cycles or more. For data analysis, mean cycle threshold (CT) values were determined for each triplicate assay and used for sample comparison, using PGK as an internal control. Individual dCT values were obtained by subtraction of the individual CT of the housekeeping gene PGK from the CT of the corresponding individual triplicate according to the ddCT method [31]. For illustration, a relative quantification (RQ) of the mean dCT values was accomplished (i.e. RQ to PGK  = 2ˆ^−dCT^), resulting in expression values relative to the mRNA expression of the internal control PGK.

### Cell culture

Rat pheochromocytoma cells (PC12 Tet-on; BD Bioscience) were cultured in 85% Dulbecco's modified eagle medium (DMEM), 10% horse serum, and 5% foetal bovine serum (all from Gibco, Carlsbad CA/USA). The isolation of *praja1* transcripts has been described previously [11]. Primer 5′-CT CGA GCC ATG AGC CAC CAG G-3′ was used to introduce an XhoI restriction site to the 5′-end of the open reading frame, allowing for in-frame cloning into the expression vectors pEGFP-C1, pCMV-HA and pTRE2-hyg (BD Bioscience). Transfections with pEGFP-Praja1.1, pEGFP-Praja1.2, or pEGFP-C1 (for acute transfection experiments), and with pTRE-EGFP-Praja1.1, pTRE-EGFP-Praja1.2, or pTRE-EGFP (for stable transfections) were done using the GeneJammer reagent (Stratagene, La Jolla CA/USA). Stably transfected PC12 cells were selected applying 500 µg/ml of G418 (Invitrogen, Carlsbad CA/USA) for 2 months and further maintained using 200 µg/ml of G418. For assessment of proliferation, neuronal differentiation, apoptosis and intracellular localization of Praja1 isoforms, acutely and stably transfected cells were allowed to adhere to collagen-IV- or PDL-coated cover slips and either further cultured under high serum conditions or in 99.6% DMEM, 0.2% horse serum, 0.2% foetal bovine serum. Neuronal differentiation of stably transfected PC12 cells was determined after supplementation of nerve growth factor (NGF, 25 ng/ml; New England Biolabs, Ipswich MA/USA) for up to 4 days. Expression of the tet-on system was achieved by parallel application of doxycycline (1 µg/ml, BD Bioscience). For analysis of neurite outgrowth, the proportion of stably transfected cells producing neurites over 25 µm in length was determined 2 and 4 days after seeding and the number of those neurites was compared between groups. For an estimation of matrix adhesion, the proportion of transfected cells showing a flattened appearance and spreading on the substrate [32] was determined in each line. The rate of apoptosis in stably transfected PC12 lines was evaluated by applying the Caspase-Glo 3/7 assay (Promega, Madison WI/USA) to cell lines after 2 days of treatment with NGF and doxycycline in the afore-mentioned manner and subsequent measurement with the Infinite M200 (Tecan, Männedorf/Switzerland). Statistical analyses of these experiments relied on one-way ANOVA and post-hoc testing.

### Immunocytochemistry

Cells were fixed in increasing concentrations (0.4–4%) of para-formaldeyhde, washed in PBS and mounted in Crystal mount (Biomeda, Foster City CA/USA). In some experiments, EGFP signals were enhanced with Ab6556 (Abcam, Cambridge/UK), which was applied at a dilution of 1∶500. Further immunocytochemical staining was done with polyclonal anti-NRAGE (Upstate, Lake Placid NY/USA) at 1∶100, polyclonal anti-Smad3 (Upstate) at 1∶200, monoclonal anti-tubulin (Sigma-Aldrich, Saint Louis MO/USA) at 1∶200, or 5 U/ml rhodamine phalloidin (Life Technologies, Eugene OR/USA). Neuronal differentiation of PC12 cells was verified by staining with anti-beta3-tubulin (Cell Signaling Technology; also named TuJ1) at 1∶200 and anti-MAP2 (Abcam; ab3096) at 1∶200, each in combination with anti-alpha-tubulin (Sigma-Aldrich) (Fig. S1). To analyse the dependence of EGFP::Praja1 localization on intact microtubules, transfected PC12 cells were treated with 100 µM colchicine (Sigma-Aldrich) before fixation and staining. Cells were routinely counterstained with DAPI to allow for an estimation of intracellular localization and to visualize pyknotic nuclei as indicators of apoptotic cell death. Cells were examined with epifluorescence microscopy and digital image capturing as well as confocal microscopy for the detection of Praja1/NRAGE and Praja1/Smad3 co-localization (Leica DMI6000, Wetzlar/Germany). Nuclear localization indices were calculated for each splice variant as an average of all EGFP-positive cells in a preparation (+1 for nuclear, 0 for equal, and −1 for predominantly cytosolic staining in each cell).

### Immunoblotting

For the analysis of endogenous Praja1 expression, 10^6^ PC12 cells were collected each at 30 min, 2 h and 3 d after stimulation with NGF. To test for polyubiquitination activity, 2*10^6^ cells were respectively collected from 2-day-old stably transfected PC12 cultures treated with NGF and doxycycline. In an additional set of experiments, polyubiquitinated proteins were accumulated in PC12 cells by 8 h pre-treatment with 1 µM lactacystin (Sigma-Aldrich). We confirmed that lactacystin treatment for 8 h or even 24 h induced cell death in less than 1.5% of the cells through staining with propidium iodide. Cells were suspended in a buffer containing 125 mM Tris (pH 6.8), 4% SDS, 20% glycerol, 10% beta-mercaptoethanol, and were denatured for 5 min at 95°C. Proteins were separated on 8% SDS-PAGE and transferred to PVDF membranes. After blocking of unspecific binding, blots were incubated with the primary antibodies: polyclonal anti-ubiquitin (Sigma-Aldrich) at a dilution of 1∶100, anti-NRAGE (Upstate) at 1∶2000, anti-Smad3 (Upstate) at 1∶1000, anti-beta-actin (Abcam) at 1∶5000, anti-alpha-tubulin (Sigma-Aldrich) at 1∶1000, anti-GFP (Abcam) at 1∶5000, or anti-HA (BD Bioscience) at 1∶1000. Rabbit anti-Praja1 serum was generated against the peptide CRSPFASTRRRWDDSE (PINEDA Antibody Service, Berlin/Germany) and used at a dilution of 1∶75 (also see Fig. S2). Signals were detected with horseradish-peroxidase-coupled secondary antibodies at a dilution of 1∶2000 to 1∶5000 (DAKO, Copenhagen/Denmark) and “ECL-plus” chemiluminescence substrate (Amersham Pharmacia Biotech, Amersham/UK), or with fluorescence-coupled anti-rabbit IRDye® 800CW and anti-mouse IRDye® 680 (both at 1∶15000) for quantification in an Odyssee scanner (LI-COR, Lincoln NE/USA). All biochemical experiments were performed at least in triplicate. During subsequent quantification, significant differences were assessed using the Student's t-test.

### Statistical analysis

Quantitative data are presented as mean +/− SEM. For comparison of two groups, a two-tailed Student's t-test has been applied. Three or more groups were compared by one- or two-way ANOVA. Homogeneity of variance was assessed using the Brown-Forsythe test. Post-hoc testing following ANOVA relied on the Tukey test for homogeneous and the Dennett-T3 test for inhomogeneous variances. An alpha below 0.05 was considered to be statistically significant.

## Results

### Expression and alternative splicing

**Figure 1:**
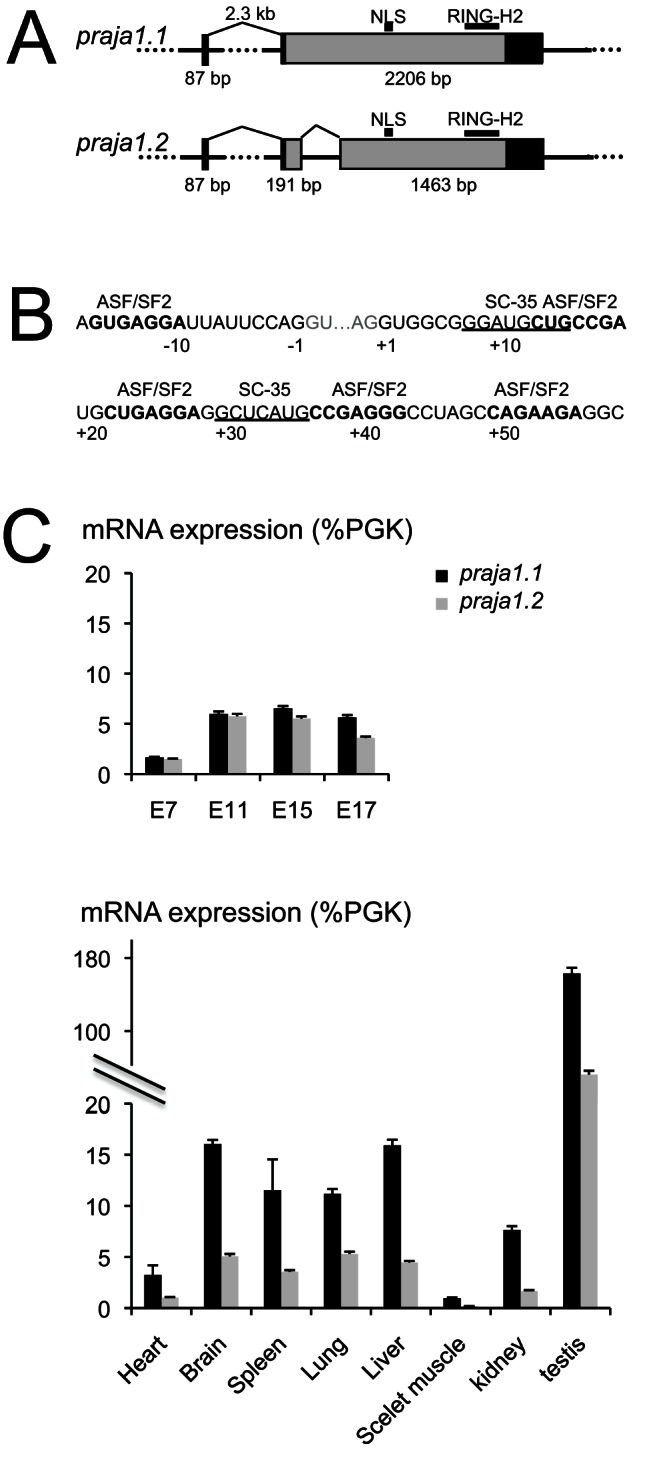
Expression of *praja1* transcript variants. (A) Two splice variants are generated from the PJA1 gene through an intron retention mechanism. Constitutive splicing of intron 1 and alternative splicing of exon 2 utilize canonical splice sites. Squares: mRNA sequence. Light grey squares: coding sequence. NLS: nuclear localization signal. Ring-H2: Ring-H2 ring finger motif. (B) Sequences surrounding splice sites in *praja1* variants and associated exonic splice enhancer motifs. Prominent SC-35 binding motifs could be identified in addition to ASF/SF2 sites. (C) *Praja1* mRNA expression is readily detectable by embryonic day E7, with a 1∶1 ratio of transcript variants *praja1.1* and *praja1.2*. In the adult mouse *praja1* mRNA expression is highest in testis, followed by brain, liver, lung, and spleen. The variants are expressed in a ratio between 2∶1 and 5∶1 in all tissues investigated (3∶1 in brain). Values are relative to the housekeeping gene phospho-glycerate kinase (PGK).

The transcript variants *praja1.1* and *praja1.2* are generated by alternative splicing of the murine *pja1* gene (Fig. 1A). ESEfinder [33] and RESCUE-ESE [34] web services identified relatively strong SC-35 ESE consensus sequences at the proximity of the splice sites (Fig. 1B). During development, a 3.5-fold increase of expression is observed between E7 and E11. Expression levels of *praja1.1* and *praja1.2* are equal until E15, after which the relative expression of *praja1.2* begins to decline. In the adult, *praja1* expression is found in all tissue samples investigated, most prominently in testis, where it is expressed at 10- to 15-fold higher levels than in brain, spleen, lung, and liver. Kidney, heart, and skeletal muscle on the other hand showed low levels of expression. In all tissues analysed, *praja1.1* is the major variant in the adult, with a 2∶1 ratio in lung, a 5∶1 ratio in kidney and skeletal muscle, and a 3∶1 ratio to *praja1.2* in all other tissues (Fig. 1C).

### Intracellular localization

**Figure 2:**
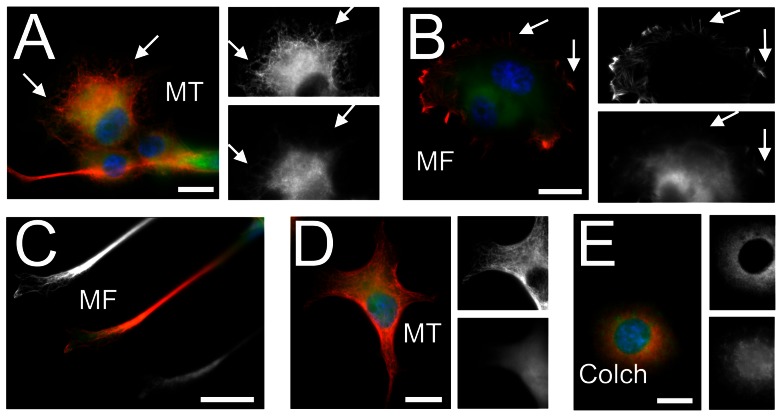
Association of Praja1 with the cytoskeleton of neuronal differentiated PC12 cells. (A) Epifluorescence microscopy reveals a filamentous intracellular distribution of EGFP::Praja1 (green), largely overlapping with microtubules (MT, red). Arrows indicate double-labelled filamentous structures in the soma. (B) Co-labelling of EGFP::Praja1 with microfilaments (MF) with phalloidin-rhodamine also indicates a partial association, in particular at filopodia (arrows) and membrane ruffles. (C) EGFP::Praja1 is also observed in outgrowing neurites and growth cones. (D) EGFP does not show a comparable filamentous distribution, and does not localize to outgrowing neurites. (E) Disruption of microtubules with colchicine (Colch) results in a diffuse intracellular localization of EGFP::Praja1. Pictures show cells transfected with the transcript variant *praja1.1*; comparable results were obtained after expression of transcript variant *praja1.2*. Sectors in black and white depict EGFP::Praja1 (lower) and the corresponding cytoskeletal element (upper). Bars: 10 µm in A, B, D and E; 5 µm in C.

**Figure 3:**
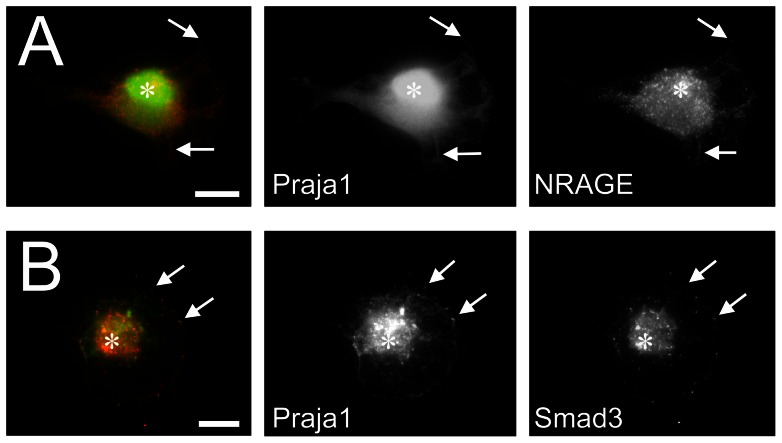
Co-localization of Praja1 with its putative substrates NRAGE and Smad3 in PC12 cells. (A) EGFP::Praja1 (green) co-localizes with NRAGE (red) in the nucleus (asterisk), at distinct positions at the membrane and along filamentous structures in the cytosol (arrows). (B) Co-localization of EGFP::Praja1 and Smad3 is also observed in the nucleus and on few points at the plasmamembrane (arrow). No difference was observed between experiments using transcript variant *praja1.1* and *praja1.2*. Bars: 10 µm.

Transfection with *praja1.1* fusion constructs generated both isoforms, Praja1.1 and Praja1.2 (henceforth referred to as Praja1.1/2), in all cell types tested (PC12, COS-7, HEK293), whereas *praja1.2* constructs produced only Praja1.2 (Fig. S2, S3). Amino acid sequence prediction suggested a mostly nuclear localization of both predicted Praja1 isoforms according to similarity with other proteins and identified the nuclear localization sequence PRRRRTM at position 292 of Praja1.1 and at position 108 of Praja1.2 (WoLF PSORT) [35]. Praja1 fusion proteins indeed displayed nuclear staining (Fig. 2, 3), but relative expression levels were higher in the cytosol than in the nucleus (nuclear index: −0.49 for EGFP::Praja1.1/2 and −0.94 for EGFP::Praja1.2 alone). In both cell lines, EGFP::Praja1 signals were further associated with microtubules (Fig. 2A) and their filamentous cytosolic distribution proved to be dependent on microtubule integrity as it disappeared upon colchicine treatment (Fig. 2E). Less frequently, EGFP::Praja1 was found in association with microfilaments, in particular at sites of neurite outgrowth and in filopodia (Fig. 2B, C). Finally, we observed a co-localization of EGFP::Praja1 with its potential substrates NRAGE and Smad3 in the nucleus, along filamentous structures in the cytosol and at distinct points at the plasmamembrane (Fig. 3). We could not observe a dependence of the intracellular localization of Praja1 on differentiation states or NGF treatment of PC12 cells.

### Neuronal differentiation of PC12

**Figure 4:**
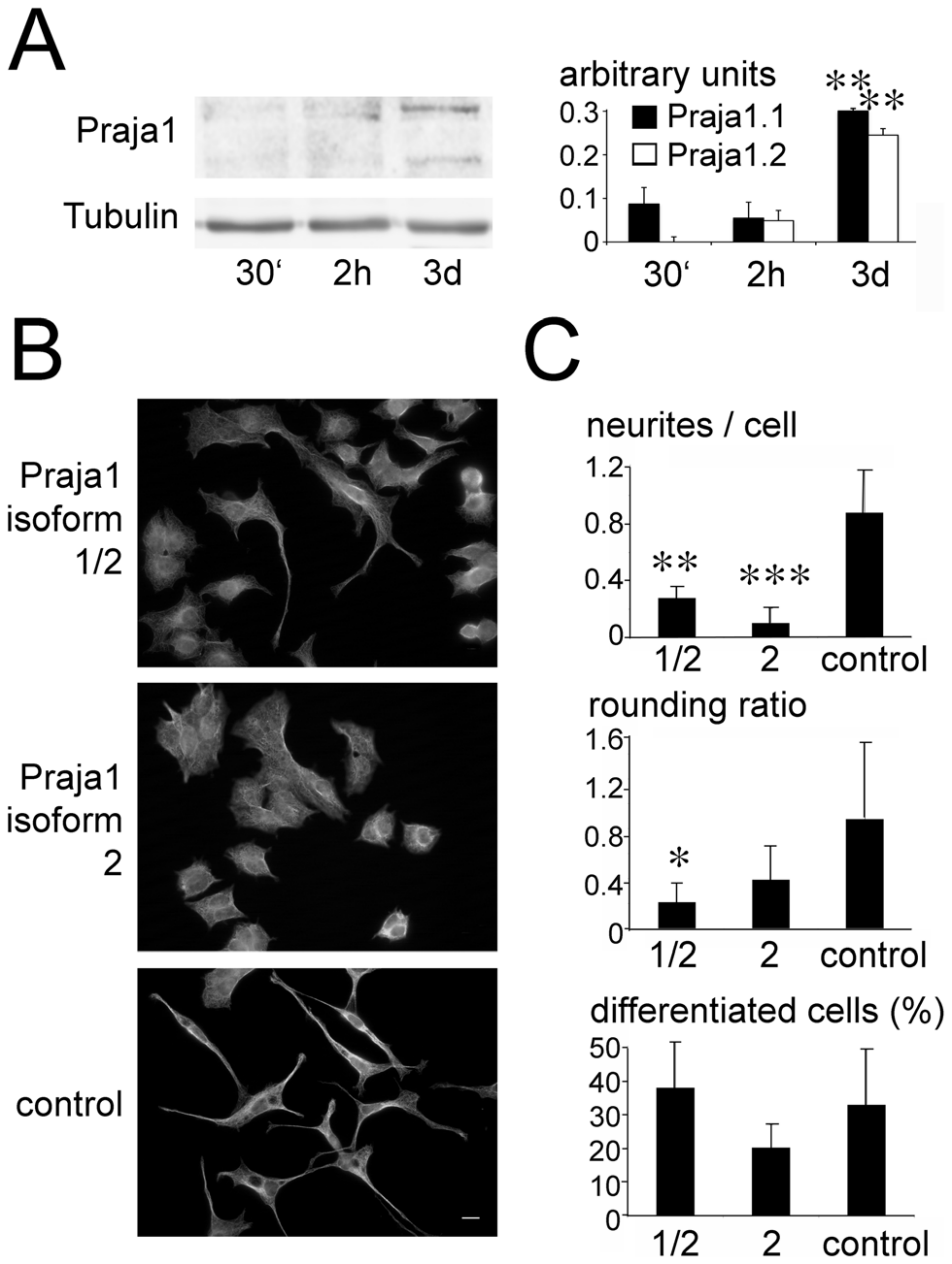
Praja1 in neuronal differentiation of PC12 cells. (A) Immunoblot analysis reveals the increased expression of endogenous Praja1 after 3 days of NGF treatment. Expression levels (normalized for tubulin) are increased more than 3-fold compared to the initial phase of outgrowth ** p<0.01, compared to 30 min after stimulation. (B) shows representative examples of PC12 cells stably expressing either EGFP::Praja1.1/2, EGFP::Praja1.2 alone, or only EGFP as control. Cells were stained with an anti-tubulin antibody to visualize their neurites. (cf. Fig. S1 with regard to the confirmation of a neuronal phenotype) (C) A reduced proportion of Praja1 overexpressing PC12 cells carries neurites of 25 µm or more compared to control cells. At the same time, the attachment of cells to the substrate is altered with Praja1.1/2 overexpressing cells showing a significantly reduced ratio of rounded to flattened cells. Praja1.2 overexpressing cells show a similar trend but fail to reach significance. In all three cell lines, a comparable proportion of cells was judged as differentiated, i.e. assuming a neuron-like morphology. All values are mean +/− SEM. * p≤0.05, ** p≤0.01, *** p≤0.001, when compared to EGFP transfected controls. Bar: 10 µm.

To address the potential function of Praja1 in neuronal differentiation, we analysed the protein expression at different stages of NGF-induced differentiation of PC12 cells. We observed low levels of Praja1.1 and Praja1.2 expression in these cells shortly after NGF stimulation, but a 3-fold increase after three days of NGF treatment (Fig. 4A).

To further study the Praja1 influence on neuronal differentiation, we established and analysed stably transfected cell lines with a doxycycline-inducible expression of EGFP-tagged Praja1. We determined neurite outgrowth as well as cell flattening with and without NGF. Spontaneous neurite formation in the absence of NGF was observed only rarely and did not exceed 1% in any of our stably transfected PC12 lines (percentage of cells with neurites for Praja1.1/2: 0.61%+/−0.41%, for Praja1.2: 0.66%+/−0.88%, for EGFP control: 0.35%+/−0.06%). Furthermore, levels of neuron specific cellular markers like beta3-tubulin and MAP2 were drastically reduced in PC12 cells without NGF treatment (Fig. S1D, E). NGF-induced neuronal differentiation on the other hand was significantly impaired in cells transfected with either transcript variant compared to control cells. Both lines showed a reduction in the number of neurites per differentiated cell (Fig. 4B, C). Moreover, cell attachment was affected by Praja1 expression, as the proportion of rounded cells was significantly reduced in PC12 cells overexpressing Praja1.1/2. A similar, yet non-significant trend was observed for Praja1.2 alone. We further tested, whether these effects were dependent on the extracellular substrate (N = 3 experiments), but similar reduction as on PDL was evident on collagen IV (p≤0.001 for neurite growth), on laminin (p≤0.01 for neurite growth, p≤0.05 for rounding), and after stimulation with the cell adhesion fc-fusion-fragment L1-Fc (p≤0.01 for neurite growth, p≤0.05 for rounding; Fig. S4). Controlling for caspase 3/7 activation in NGF-differentiated cells we could not observe any sign for an altered rate of apoptosis (mean relative luminescence units for PC12 cells expressing Praja1.1/2: 69869.71+/−29127.14, Praja1.2: 43128.50+/−12956.53, control: 72677.00+/−32428.92).

### Ubiquitination

**Figure 5:**
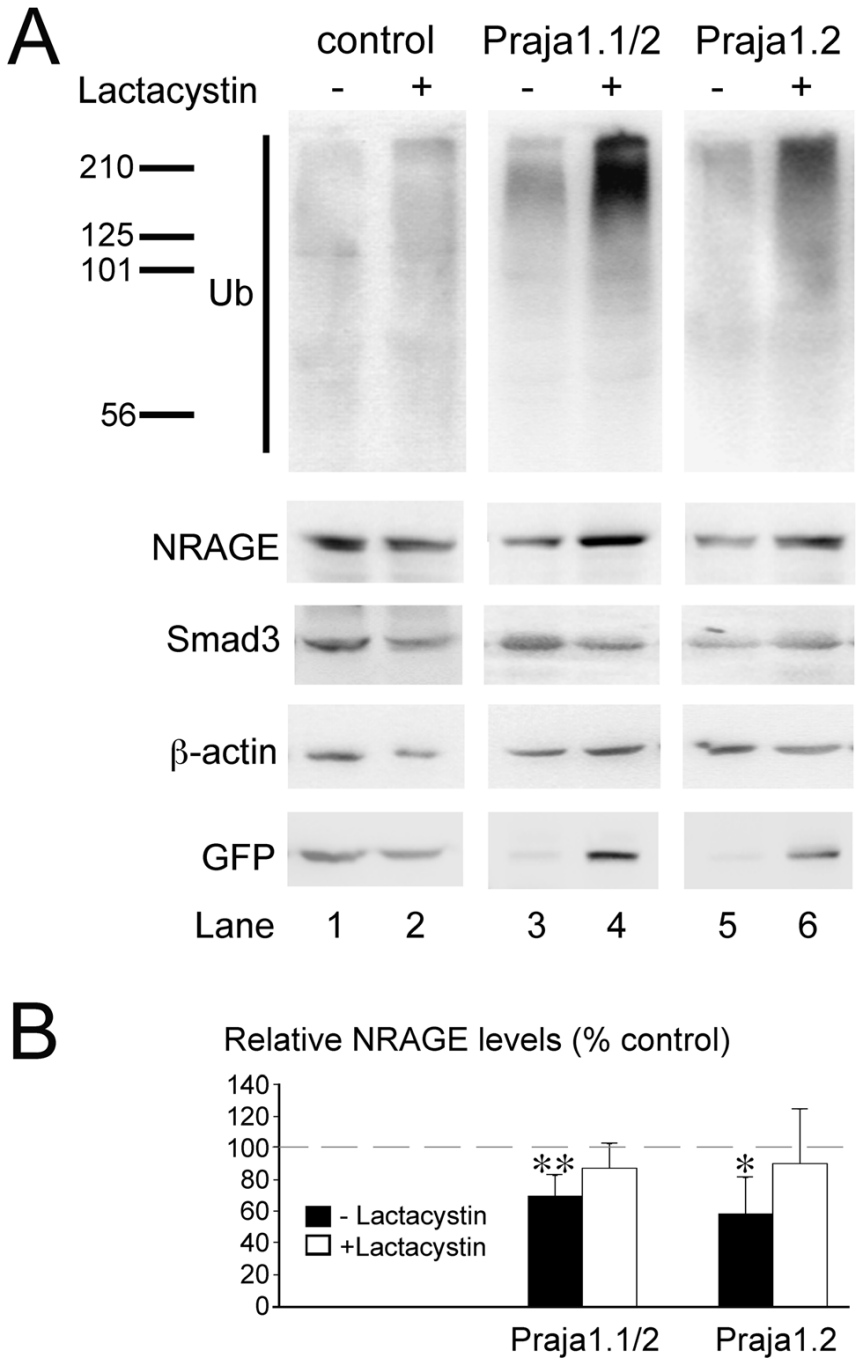
Polyubiquitination and NRAGE expression levels in PC12 cells. (A) Immunoblot analysis reveals an increase of total protein ubiquitination in cells expressing EGFP::Praja1.1/2 (lanes 3&4) or only EGFP::Praja1.2 (lanes 5&6) compared to EGFP transfected controls (lanes 1&2). However, this effect is only apparent following blockade of proteasomal degradation and subsequent accumulation of ubiquitinated proteins with lactacystin (lanes 4&6). Endogenous NRAGE is reduced by Praja1 overexpression (lanes 3&5) in a lactacystin sensitive manner, but no change in Smad3 expression levels is apparent. Expression levels of EGFP::Praja1.1 and EGFP::Praja1.2 are strongly increased following lactacystin, in agreement with an ability to self-regulate activity through auto-ubiquitination, as implied previously [36], [37]. (B) Quantitative analysis of Western blots (N = 5) showed a significant reduction of NRAGE upon Praja1 overexpression that was prevented by treatment of cells with the proteasome inhibitor lactacystin. Values presented are normalized to control transfected cells undergoing the same lactacystin treatment. * p≤0.05, ** p≤0.01.

Ubiquitination and NRAGE expression were assessed in the presence and absence of the proteasome inhibitor lactacystin. Increased polyubiquitination compared to controls was detected in PC12 cells expressing either both, EGFP::Praja1.1 and EGFP::Praja1.2, or only EGFP::Praja1.2 (Fig. 5). However, these effects were only seen following treatment with the proteasome inhibitor lactacystin, which led to a more pronounced accumulation of polyubiquitinated proteins in these cells. Expression of NRAGE was significantly reduced in both Praja1 overexpressing cell lines, but these changes were prevented by the treatment with lactacystin. Smad3 levels, tested to control for the specificity of the observed effects, were not affected by the Praja1 expression. In accordance with earlier hypothesis of an auto-regulation of Praja1 through the ubiquitin/proteasome pathway [36], [37], EGFP::Praja1.1 and EGFP::Praja1.2 accumulated following lactacystin treatment.

## Discussion

The ubiquitin ligase Praja1 has been implicated in liver development [9], gastric cancer [37], [38] and plasticity in the adult nervous system [11]. However, its precise cellular function is still far from understood. In the current study, we describe the expression of *praja1* mRNA in different mouse tissues and developmental stages and investigate Praja1 effects on NGF-induced neuronal differentiation. We demonstrate that Praja1, likely through polyubiquitination and degradation of the multifactor signalling molecule NRAGE, suppresses the neurite outgrowth in PC12 cells.

Two splice variants of murine *praja1* have been identified, which appear to be generated through a differential intron retention mechanism using canonical U2 splice donor and splice acceptor sites located within the second exon. *In vivo*, we found that the ratio of *praja1.1* to *praja1.2* changes from roughly 1∶1 in early development to 3:1 in most adult tissues including the brain, suggesting an enhanced developmental expression and function of *praja1.2*. We generated stably transfected PC12 cell lines using both transcript variants in order to study their role in neuronal differentiation. Cells transfected with *praja1.1* constructs overexpressed both, Praja1.1 and Praja1.2, in a stoichiometry similar to the endogenous expression in the adult brain, whereas cells transfected with *praja1.2* generated only the primarily developmental form Praja1.2.

However, evidence from our experiments suggests a generally comparable function of both Praja1 isoforms. In both cell lines, Praja1 was found to predominate in the cytosol, frequently in association with microtubules and filopodial actin filaments. This intracellular distribution, together with the partial co-localization with NRAGE and Smad3 at these cytoskeletal elements, at few, but distinct positions at the cell membrane, and in the nucleus may suggest a role in cytoskeletal rearrangement and/or the signalling of these cytoskeleton-associated factors to the nucleus. On day three of NGF-induced outgrowth stimulation, expression of both isoforms of endogenous Praja1 was increased, suggesting a role during late stages of differentiation. Overexpression of either Praja1.1/2 or of Praja1.2 alone, however, resulted in a profound reduction of neurite outgrowth and in an increase of cell spreading.

Previously, Praja1 has been shown to precipitate NRAGE in a GST pull-down and to occur in a complex with NRAGE and Msx2 in HEK293 cells [14], [39]. In addition, NRAGE has been reported to stimulate neuronal differentiation and neurite outgrowth in PC12 cells [29], [40]. We therefore hypothesized that Praja1-mediated ubiquitination and proteasomal degradation of NRAGE may explain its effect on neuritogenesis. Indeed, the overexpression of either one of the Praja1 variants induced a lactacystin-sensitive reduction of endogenous NRAGE in PC12 cells. Our current data thus support those reported by Reddy and co-workers, who observed increased neuronal differentiation upon stable overexpression of NRAGE in PC12 cells [29]. Also, overexpression of the p75^NTR^-associated MAGE family members necdin and MAGE-H1 [24], [41] has been shown to increase NGF-induced and TrkA-dependent neurite growth in PC12 cells in co-operation with endogenous NRAGE [40]. On the other hand, Feng *et al*. have reported opposite effects, seeing a reduction of neuronal differentiation and associated ERK signalling after NRAGE overexpression and an increased NGF-induced ERK activation after knocking down NRAGE [28]. Moreover, previous studies have demonstrated that p75^NTR^-mediated apoptosis – rather than differentiation – is facilitated by NRAGE in various cell types including PC12 [24], [27] and that Praja1 targets various anti-apoptotic factors [13]. Therefore, it is important to note that overexpression of Praja1 in PC12 cells, in contrast to COS-7 cells, which showed cell rounding, formation of microspikes and pyknotic nuclei (Fig. S3, S5), was not associated with an induction of apoptosis. Praja1, by controlling the level of NRAGE and other intracellular signalling factors [13], may thus contribute to controlling the delicate balance between p75 and TrkA receptor function in differentiating PC12 cells [42].

How might these findings translate to the developing and mature nervous system? NRAGE has been shown to modulate the function of cell adhesion molecules and their interaction with the cytoskeleton, as well as signalling to the nucleus [19], [39], [43], [44]. Praja1 and NRAGE, like necdin [45], [46], are expressed early in development and in a large number of adult tissues including the brain [11], [30]. The up-regulation of Praja1 expression between embryonic days 7 and 11 observed in this study coincides with an increase of NRAGE expression in the mouse embryo [30]. NRAGE is indispensable for the interaction of necdin with Dlx2 and critically involved in the differentiation of GABAergic forebrain neurons [43]. In addition, NRAGE appears to be involved in neuronal differentiation processes independently of necdin, which is not endogenously expressed in PC12 cells [38], [39], [46]. Necdin, NRAGE, and Praja1 also exist at high levels in the adult hippocampus and amygdala [11], [23], [45], and changes in the expression or interaction of these signalling molecules may contribute to neural plasticity and information storage in these structures. Furthermore, previous studies have shown that Praja1 can also modulate the NRAGE-mediated activation of the Dlx5 transcription factor complex in HEK293 cells through direct interaction and without the need for proteasomal degradation of NRAGE [38].

## Conclusions

Previous studies have shown that NGF-induced neurite outgrowth in PC12 cells coincides with a modulation of intracellular ubiquitination activity [47] and that proteasome inhibitors influence neurite outgrowth in PC12 cells and primary neurons [47], [48]. Yet, former results regarding this influence have been contradictory. Our data indicate that Praja1-mediated ubiquitination mechanisms, by decreasing expression levels of the multifactor-signalling molecule NRAGE, negatively regulate neurite growth. The induction of Praja1 during NGF-induced differentiation suggests that these mechanisms may be involved in the termination and fine-tuning of neurite formation.

## Supporting Information

Figure S1 **Confirmation of a neuronal phenotype.** PC12 cells of each stably transfected line were stained with antibodies against the neuron-specific markers beta3-tubulin (also called TuJ1; red) or MAP2 (red), each in combination with DAPI (cyan) and anti-alpha-tubulin (blue). (A) through (C) show the separate and merged stainings for cells overexpressing Praja1.1/2 or Praja1.2 and control cells after NGF treatment. Arrows indicate the expression of beta3-tubulin in neurites particularly at the growth tip. (D) and (E) exemplify the lack of spontaneous differentiation in absence of NGF. Neurites are not seen and levels of beta3-tubulin (D) or MAP2 (E) are almost undetectable. Cells overexpressing Praja1 isoforms showed equal results. (F) and (G) present the MAP2 labelling of Praja1.1/2 expressing cells and control cells after NGF treatment, which, in essence, are equivalent to staining of beta3-tubulin. Bars: 25 µm (which has served as threshold in our experiments).
(TIF)

Figure S2 **Specificity of anti-Praja1 serum.** The expression of both Praja1.1 and Praja1.2 from HA-tagged *praja1.1* (lane 3) is detected in HEK293T cells using Praja-specific serum. The apparent molecular weight of ca. 95 kD and 65 kD, respectively, differs clearly from the predicted molecular weight of the two isoforms but is in agreement with the previously reported reduced migration of Praja1.2 in PAGE [9]. The expression level ratio of isoforms is 3∶1, resembling the ratio in differentiated PC12 cells. Detection with anti-HA confirms the specificity of the Praja1 antiserum (lane 6). An unspecific signal is detected at ca. 130 kD in all lanes, including MOCK control (lanes 1&4) and pCMV-HA transfected control cells (lanes 2&5). Detection of anti-tubulin serves as loading control.
(TIF)

Figure S3 **Praja1-induced polyubiquitination and changes of NRAGE levels in COS-7 cells.** Immunoblot analysis shows an increase of total protein ubiquitination and reduction of endogenous NRAGE in cells overexpressing HA-tagged Praja1.1 and Praja1.2 (lane 2) or HA-tagged Praja1.2 alone (lane 3) compared to mock controls (lane 1). At the same time, levels of NRAGE are reduced. Smad3 levels are low and unchanged upon Praja1 expression (data not shown). These results are also confirmed in an independent set of experiments using EGFP-tagged transcript variants (data not shown). The lower panel demonstrates the expression of Praja1 isoforms; beta-actin serves as loading control.
(TIF)

Figure S4 **Substrate independence of Praja effects.** (A) Reduced growth of neurites of ≥25 µm is observed in cells overexpressing Praja1.1/2, regardless of the substrate used. (B) Cell rounding is reduced on all tested substrates except for collagen IV. (C) However, the overall proportion of differentiated cells, showing a neuron-like morphology, is generally not affected by Praja1 overexpression, except on laminin (but not on laminin/PDL). Cells were counted as differentiated when they showed filopodia of ≥5 µm and/or neurites of ≥25 µm. Significance levels were assessed by using Student's t-test. *** p≤0.001, ** p≤0.01, * p≤0.05.
(TIF)

Figure S5 **Induction of apoptosis in COS-7 cells through Praja1 expression.** (A) COS-7 cells acutely transfected with *praja1.1* or *praja1.2* display cell rounding and microspike formation (arrow), and develop pyknotic nuclei, indicating an induction of apoptosis. EGFP and HA fusion proteins are equally effective in inducing this phenotype (data not shown), whereas transfection with control vectors has no such effect. (B) Quantitative analysis of cell morphology indicates differences in the effectiveness of Praja1 isoforms to induce this change. On the one hand, cell rounding is similarly induced by EGFP::Praja1.1/2 (cell size 410+/−235 µm^2^, 14.3% spreading cells) and EGFP::Praja1.2 (cell size 245+/−115 µm^2^, 18.2% spreading cell), as compared to EGFP transfected controls (cell size 660+/−459 µm^2^, 53.9% spreading cells) or non-transfected cells within the samples (cell size 909-1071 µm^2^, 82.2%–89.8% spreading cells). On the other hand, the incidence of microspike formation decreases from combined overexpression of EGFP::Praja1.1 and EGFP::Praja1.2 (78.6%) to EGFP::Praja1.2 alone (54.5%). Microspike forming cells in EGFP controls (7.7%) are similar to those in non-transfected cells (2.2%–5.9%). The occurrence of pyknotic nuclei confirms the induction of apoptosis in 51.6% of *praja1.1* and 60.0% of *praja1.2* transfected cells. Bars: 20 µm. Significance levels were assessed by a two-way ANOVA. ** p≤0.01 compared to control transfected cells.
(TIF)

Procedure S1 **Supplemental experimental procedures.**
(DOC)
