# Supplementary figures and images for: The Ubiquitin Ligase Praja1 Reduces NRAGE Expression and Inhibits Neuronal Differentiation of PC12 Cells
Source: PLoS One. 2013 May 22;8(5):e63067. doi: 10.1371/journal.pone.0063067 (PMC3661586; doi:10.1371/journal.pone.0063067)

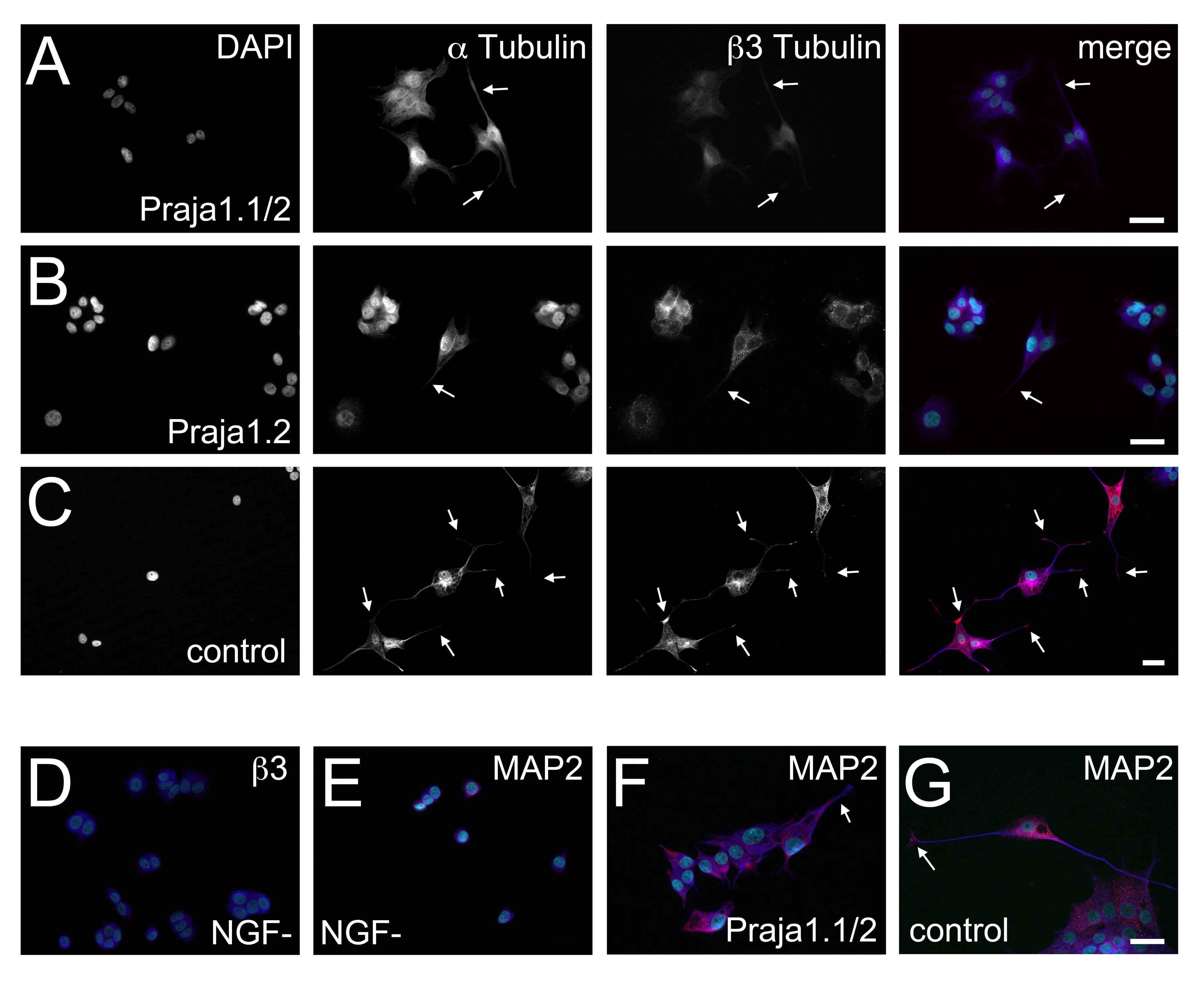

Supplement: Figure S1 — Confirmation of a neuronal phenotype. PC12 cells of each stably transfected line were stained with antibodies against the neuron-specific markers beta3-tubulin (also called TuJ1; red) or MAP2 (red), each in combination with DAPI (cyan) and anti-alpha-tubulin (blue). (A) through (C) show the separate and merged stainings for cells overexpressing Praja1.1/2 or Praja1.2 and control cells after NGF treatment. Arrows indicate the expression of beta3-tubulin in neurites particularly at the growth tip. (D) and (E) exemplify the lack of spontaneous differentiation in absence of NGF. Neurites are not seen and levels of beta3-tubulin (D) or MAP2 (E) are almost undetectable. Cells overexpressing Praja1 isoforms showed equal results. (F) and (G) present the MAP2 labelling of Praja1.1/2 expressing cells and control cells after NGF treatment, which, in essence, are equivalent to staining of beta3-tubulin. Bars: 25 µm (which has served as threshold in our experiments). (TIF) [file pone.0063067.s001.tif]

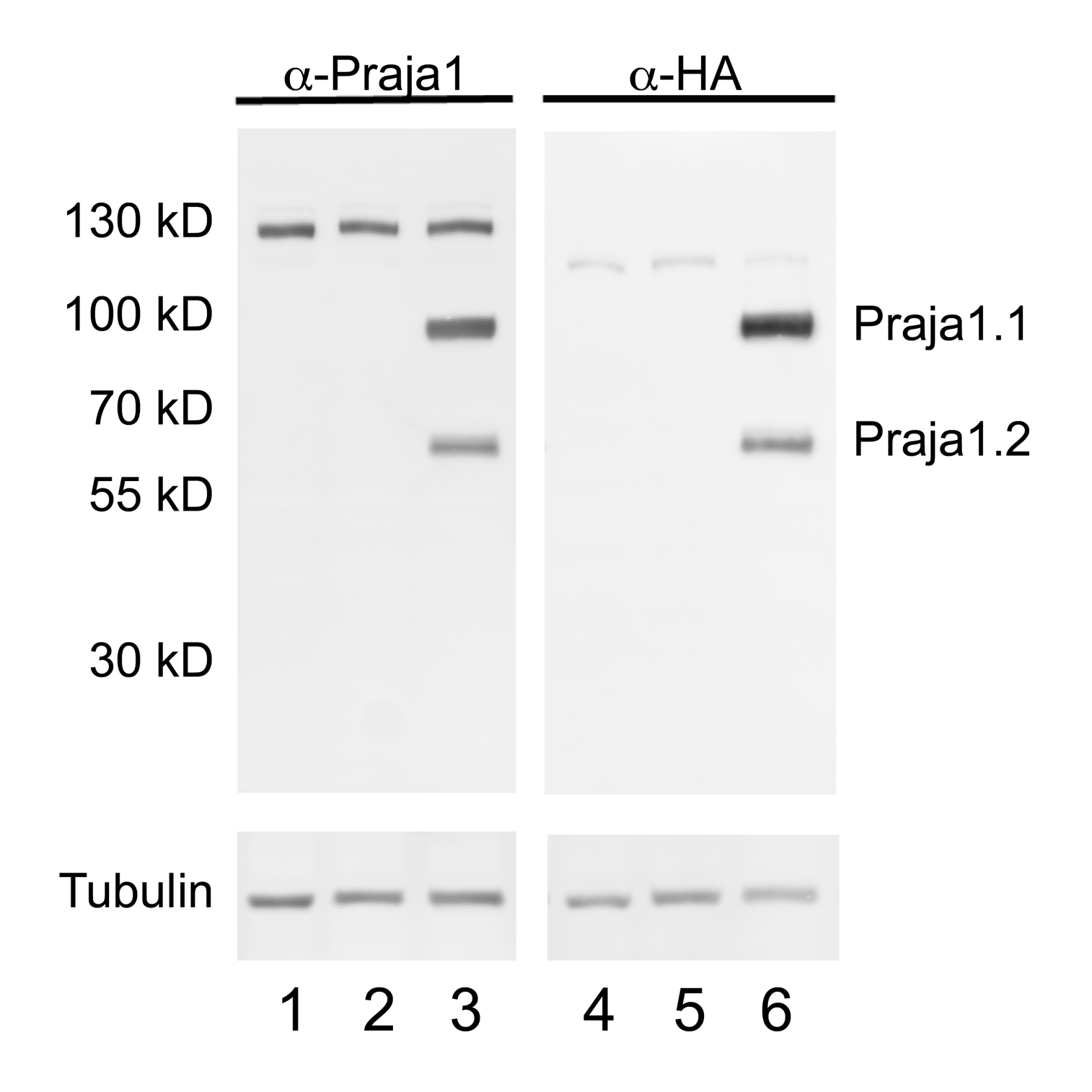

Supplement: Figure S2 — Specificity of anti-Praja1 serum. The expression of both Praja1.1 and Praja1.2 from HA-tagged praja1.1 (lane 3) is detected in HEK293T cells using Praja-specific serum. The apparent molecular weight of ca. 95 kD and 65 kD, respectively, differs clearly from the predicted molecular weight of the two isoforms but is in agreement with the previously reported reduced migration of Praja1.2 in PAGE [9]. The expression level ratio of isoforms is 3∶1, resembling the ratio in differentiated PC12 cells. Detection with anti-HA confirms the specificity of the Praja1 antiserum (lane 6). An unspecific signal is detected at ca. 130 kD in all lanes, including MOCK control (lanes 1&4) and pCMV-HA transfected control cells (lanes 2&5). Detection of anti-tubulin serves as loading control. (TIF) [file pone.0063067.s002.tif]

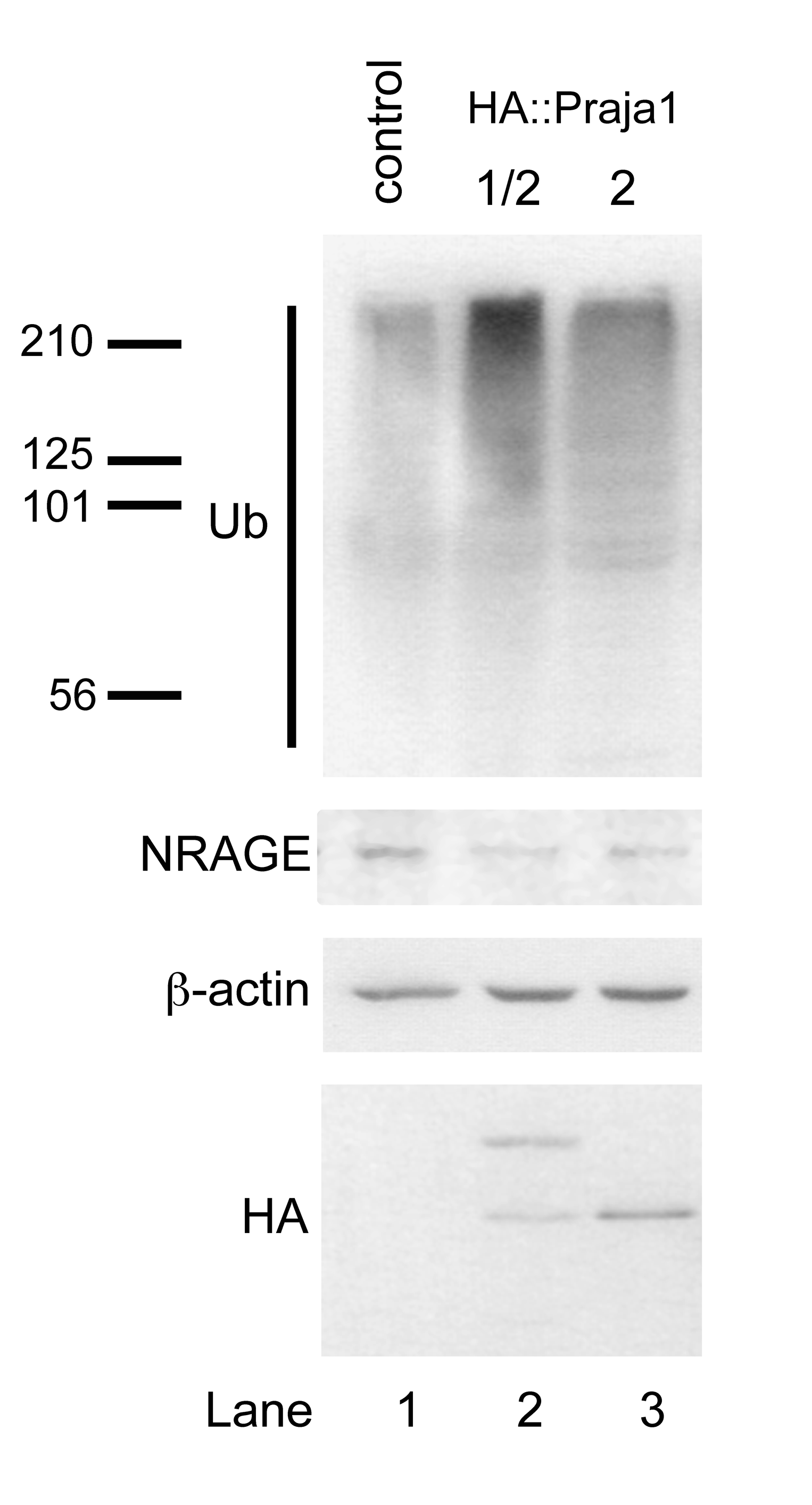

Supplement: Figure S3 — Praja1-induced polyubiquitination and changes of NRAGE levels in COS-7 cells. Immunoblot analysis shows an increase of total protein ubiquitination and reduction of endogenous NRAGE in cells overexpressing HA-tagged Praja1.1 and Praja1.2 (lane 2) or HA-tagged Praja1.2 alone (lane 3) compared to mock controls (lane 1). At the same time, levels of NRAGE are reduced. Smad3 levels are low and unchanged upon Praja1 expression (data not shown). These results are also confirmed in an independent set of experiments using EGFP-tagged transcript variants (data not shown). The lower panel demonstrates the expression of Praja1 isoforms; beta-actin serves as loading control. (TIF) [file pone.0063067.s003.tif]

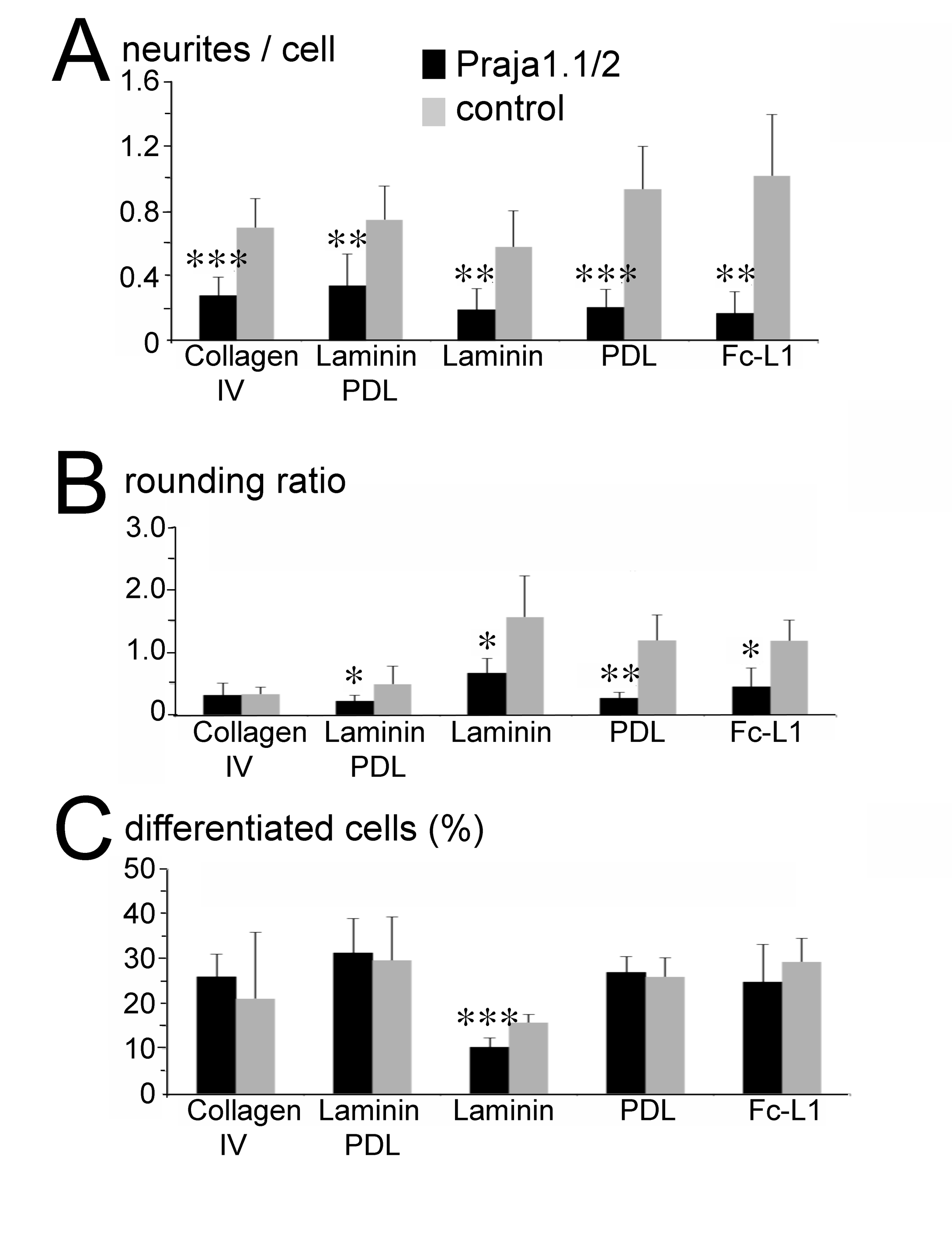

Supplement: Figure S4 — Substrate independence of Praja effects. (A) Reduced growth of neurites of ≥25 µm is observed in cells overexpressing Praja1.1/2, regardless of the substrate used. (B) Cell rounding is reduced on all tested substrates except for collagen IV. (C) However, the overall proportion of differentiated cells, showing a neuron-like morphology, is generally not affected by Praja1 overexpression, except on laminin (but not on laminin/PDL). Cells were counted as differentiated when they showed filopodia of ≥5 µm and/or neurites of ≥25 µm. Significance levels were assessed by using Student's t-test. *** p≤0.001, ** p≤0.01, * p≤0.05. (TIF) [file pone.0063067.s004.tif]

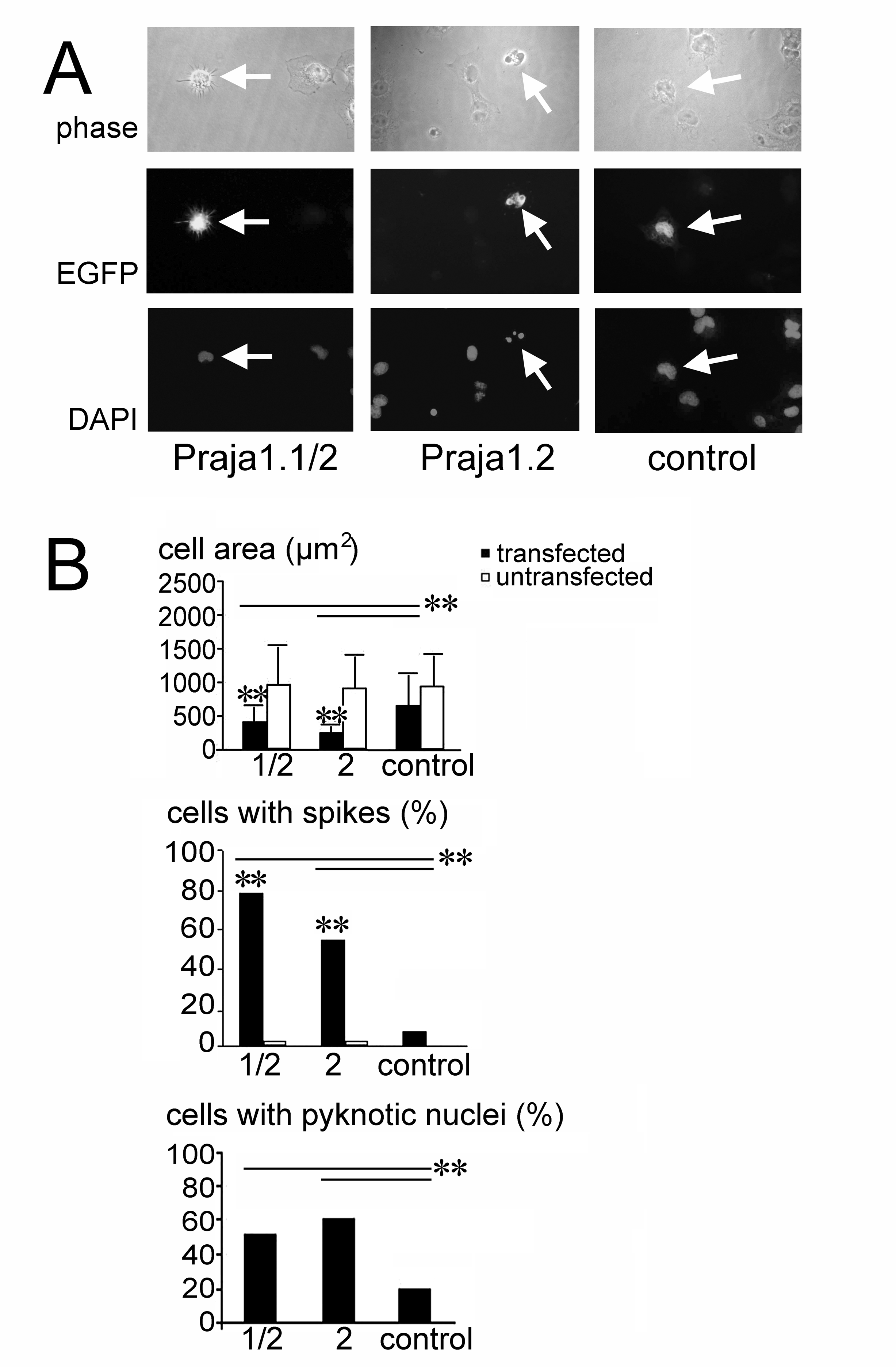

Supplement: Figure S5 — Induction of apoptosis in COS-7 cells through Praja1 expression. (A) COS-7 cells acutely transfected with praja1.1 or praja1.2 display cell rounding and microspike formation (arrow), and develop pyknotic nuclei, indicating an induction of apoptosis. EGFP and HA fusion proteins are equally effective in inducing this phenotype (data not shown), whereas transfection with control vectors has no such effect. (B) Quantitative analysis of cell morphology indicates differences in the effectiveness of Praja1 isoforms to induce this change. On the one hand, cell rounding is similarly induced by EGFP::Praja1.1/2 (cell size 410+/−235 µm2, 14.3% spreading cells) and EGFP::Praja1.2 (cell size 245+/−115 µm2, 18.2% spreading cell), as compared to EGFP transfected controls (cell size 660+/−459 µm2, 53.9% spreading cells) or non-transfected cells within the samples (cell size 909-1071 µm2, 82.2%–89.8% spreading cells). On the other hand, the incidence of microspike formation decreases from combined overexpression of EGFP::Praja1.1 and EGFP::Praja1.2 (78.6%) to EGFP::Praja1.2 alone (54.5%). Microspike forming cells in EGFP controls (7.7%) are similar to those in non-transfected cells (2.2%–5.9%). The occurrence of pyknotic nuclei confirms the induction of apoptosis in 51.6% of praja1.1 and 60.0% of praja1.2 transfected cells. Bars: 20 µm. Significance levels were assessed by a two-way ANOVA. ** p≤0.01 compared to control transfected cells. (TIF) [file pone.0063067.s005.tif]
