# Supplementary material for: The Ubiquitin Ligase Praja1 Reduces NRAGE Expression and Inhibits Neuronal Differentiation of PC12 Cells
Source: PLoS One. 2013 May 22;8(5):e63067. doi: 10.1371/journal.pone.0063067 (PMC3661586; doi:10.1371/journal.pone.0063067)
Supplement: Procedure S1 — Supplemental experimental procedures. (DOC) [file pone.0063067.s006.doc]

African green monkey kidney fibroblast (COS-7) cells were cultured in 90 % Dulbeccos’s modified eagle medium (DMEM) and 10 % foetal bovine serum (both from Gibco). Transfections with pCMV-HA-Praja1.1, pCMV-HA-Praja1.2, or pCMV-HA were performed using the GeneJammer reagent (Stratagene) according to the manufacturer’s instructions. Cells were allowed to adhere to collagen-I-coated cover slips for 2 h before transfection and were analysed 48 h later. Appropriate expression of the splice variants upon transfection was confirmed through real-time PCR and Western blot analysis. Cell rounding, formation of microspikes and occurrence of pyknotic nuclei in DAPI stains were analysed as indicators of apoptosis using light and epifluorescence microscopy.
